# Associations of anthropometric markers with serum metabolites using a targeted metabolomics approach: results of the EPIC-potsdam study

**DOI:** 10.1038/nutd.2016.23

**Published:** 2016-06-27

**Authors:** U Bachlechner, A Floegel, A Steffen, C Prehn, J Adamski, T Pischon, H Boeing

**Affiliations:** 1 Department of Epidemiology, German Institute of Human Nutrition Potsdam-Rehbruecke, Nuthetal, Germany; 2 Institute of Experimental Genetics, Helmholtz Center Munich, German Research Center for Environmental Health, Neuherberg, Germany; 3 German Center for Diabetes Research, Neuherberg, Germany; 4 Institute of Experimental Genetics, Technical University of Munich, Freising-Weihenstephan, Germany; 5 Molecular Epidemiology Group, Max Delbrück Center for Molecular Medicine (MDC), Berlin-Buch, Germany

## Abstract

**Background/Objectives:**

The metabolic consequences of type of body shape need further exploration. Whereas accumulation of body mass in the abdominal area is a well-established metabolic risk factor, accumulation in the gluteofemoral area is controversially debated. We evaluated the associations of anthropometric markers of overall body mass and body shape with 127 serum metabolites within a sub-sample of the European Prospective Investigation into Cancer and Nutrition (EPIC)-Potsdam cohort.

**Subjects/Methods:**

The cross-sectional analysis was conducted in 2270 participants, randomly drawn from the EPIC-Potsdam cohort. Metabolites were measured by targeted metabolomics. To select metabolites related with both waist circumference (WC) (abdominal subcutaneous and visceral fat) and hip circumference (HC) (gluteofemoral fat, muscles and bone structure) correlations (*r*) with body mass index (BMI) as aggregating marker of body mass (lean and fat mass) were calculated. Relations with body shape were assessed by median metabolite concentrations across tertiles of WC and HC, mutually adjusted to each other.

**Results:**

Correlations revealed 23 metabolites related to BMI (*r*⩾I0.20 I). Metabolites showing relations with BMI were showing similar relations with HC adjusted WC (WC_HC_). In contrast, relations with WC adjusted HC (HC_WC_) were less concordant with relations of BMI and WC_HC_. In both sexes, metabolites with concordant relations regarding WC_HC_ and HC_WC_ included tyrosine, diacyl-phosphatidylcholine C38:3, C38:4, lyso-phosphatidylcholine C18:1, C18:2 and sphingomyelin C18:1; metabolites with opposite relations included isoleucine, diacyl-phosphatidylcholine C42:0, acyl–alkyl-phosphatidylcholine C34:3, C42:4, C42:5, C44:4 and C44:6. Metabolites specifically related to HC_WC_ included acyl–alkyl-phosphatidylcholine C34:2, C36:2, C38:2 and C40:4, and were solely observed in men. Other metabolites were related to WC_HC_ only.

**Conclusions:**

The study revealed specific metabolic profiles for HC_WC_ as marker of gluteofemoral body mass differing from those for BMI and WC_HC_ as markers of overall body mass and abdominal fat, respectively. Thus, the study suggests that gluteofemoral mass may have less-adverse metabolic implications than abdominal fat.

## Introduction

Overweight and obesity are related to metabolic disorders (for example, impaired fasting glucose, impaired glucose tolerance, hypertriglyceridemia, type-2-diabetes).^1^ A major determinant of weight-associated metabolic disorders is the type of body shape. The commonly referred ‘apple' shape, characterized by predominating abdominal fat accumulation, is supposed to pose greater health risks than the ‘pear' shape, characterized by predominating gluteofemoral fat accumulation.^2^ Health risks related to abdominal fat are mainly driven by the visceral fat compartment, whose expansion is a well-established metabolic risk factor.^3, 4^ Expansion of subcutaneous fat, such as gluteofemoral fat, presumably does not have metabolically adverse effects, but is supposed to act protective.^5, 6^

Overweight and obesity are classified by body mass index (BMI), a simple population level measure, which is defined as one's weight in kilograms divided by the square of one's height in meters. Consequently, BMI is an aggregating marker of body mass covering lean mass (mainly including muscles, bones and water) and fat mass. Using BMI to classify excess body fat is based on the assumption that at a given height, higher weight is associated with increased fatness.^7^ To assess body shape, circumferences such as waist circumference (WC) and hip circumference (HC) are commonly used as surrogate measures. Whereas WC comprises mainly abdominal subcutaneous and visceral fat, HC comprises gluteofemoral fat, muscles and bone structure (pelvic width). Furthermore, it must be borne in mind that anthropometric measures are generally affected by an individual's body frame. Even though circumferences can only provide rough estimates of one's true body composition, WC is supposed to be the most effective single measure for visceral fat.^8, 9, 10^ HC generally reflects gluteofemoral body mass, but it is not able to differentiate between parts attributed to fat, muscles or bone structure. Moreover, parts may clearly differ between men and women. However, in both sexes HC highly correlates with subcutaneous fat.^10^

Excess weight affects the whole body and apparently involves metabolic changes.^11, 12, 13, 14^ The metabolomic approach has already been used to identify metabolites involved in overweight and obesity^11, 15, 16, 17, 18, 19^ and weight-associated metabolic disorders, for example, pre-diabetes (that is, impaired fasting glucose and/or impaired glucose tolerance)^12, 20, 21, 22, 23^ and clinically manifest type-2-diabetes.^24, 25, 26^ The metabolic state particularly related with body shape is not fully understood. It is assumed that the accumulation of visceral fat has an important etiological role for weight-associated metabolic disorders,^27, 28^ whereas the accumulation of gluteofemoral body mass was found to be associated with more favorable metabolic profiles, when abdominal fat was taken into account.^29, 30^ In the present study, we aimed to gain a broader understanding of the metabolic state related to body mass in general and body shape in particular. Therefore, we evaluated the associations of anthropometric markers of overall body mass and body shape with 127 targeted metabolites in chronic disease-free individuals within a sub-sample of the European Prospective Investigation into Cancer and Nutrition (EPIC)-Potsdam cohort.

## Subjects and methods

### Ethics statement

The study was approved by the ethics committee of the Medical Association of the State of Brandenburg. A written informed consent from all participants was obtained.

### Study population

The EPIC-Potsdam cohort is part of the ongoing multicenter EPIC study and comprises 27 548 participants from the general population of Potsdam and surroundings, recruited between 1994 and 1998.^31^

The present analysis was conducted in a randomly drawn sub-sample (*n*=2500) from the EPIC-Potsdam study population with blood samples (*n*=26 448). Participants, mainly aged 35–65 years, filled out socio-demographic and lifestyle questionnaires, a validated food-frequency questionnaire and completed an interview on medical history. Participants additionally had anthropometric measures and a blood sample taken by trained staff.^32, 33^ In brief, 30 ml of blood were collected and immediately processed according to a strict protocol.^34^ The blood was fractionated into serum, plasma, buffy coat and erythrocytes, aliquoted into straws of 0.5 ml, and stored in liquid nitrogen tanks at −196 °C until analysis.

In this cross-sectional study we excluded participants with missing anthropometric measurements, missing blood samples or biomarker measurement, missing covariate information and participants with prevalent chronic diseases. Thus, the analytical sample included 2270 individuals.

### Measurement of anthropometry

Anthropometry was measured according to standardized procedures by trained staff as described earlier.^35^ Weight was obtained without shoes in light clothing to the nearest of 0.1 kg. Height and circumferences were measured to the nearest of 0.1 cm. WC was taken midway between the iliac crest and the lower ribs. HC was measured over the buttocks. BMI was calculated as weight in kilograms divided by the square of height in meters.

### Measurement of serum metabolites

Metabolomic measurements were performed in the Genome Analysis Center at the Helmholtz Center Munich by electrospray ionization-tandem mass spectrometry using the Absolute*IDQ* p150 kit (BIOCRATES Life Sciences AG, Innsbruck, Austria). Sample preparation has been described in detail previously.^36, 37^ In brief, 10 μl of serum were pipetted onto a filter in a 96-well sandwich plate, containing stable isotope-labeled internal standards. Amino acids (AA) were derivated with 5% phenylisothiocyanate reagent. After extraction of metabolites and internal standards with 5 mm ammonium acetate in methanol, the solution was centrifuged through a filter membrane and diluted with mass spectrometry running solvent. Final extracts were analyzed and 163 metabolites quantified simultaneously, including free carnitine, 40 acylcarnitines, 14 AA (13 proteinogenic+ornithine), one hexose (sum of six carbon monosaccharides without distinction of isomers), 92 glycerophospholipids (15 lyso-phosphatidylcholines (lysoPC), 77 diacyl (aa)- and acyl–alkyl (ae)- phosphatidylcholines (PC)) and 15 sphingomyelins (SM). Internal standards were used to calculate metabolite concentrations. Fatty-acid side chains were abbreviated C*x*:y where *x* represented the number of carbon atoms and *y* the number of double bonds. The acylcarnitines were derivates of carnitine (C0) with one fatty-acid bond (C*x*:*y*). The prefix ‘lyso' indicated a single fatty-acid side chain. Concentrations are reported in μm. The median analytical variance of EPIC-Potsdam samples was 7.3% within-plate coefficient of variation and 11.3% between-plates coefficient of variation.^38^ After exclusion of metabolites below the limit of detection (*n*=30) and those with high analytical variance in our samples (*n*=6), 127 metabolites (17 acylcarnitines, 14 AA, one hexose, 81 glycerophospholipids and 14 SM) were remaining for the present analysis.

### Statistical analysis

Descriptive statistics were calculated as mean and s.d. for continuous variables and percentage for categorical variables.

As most of the metabolite concentrations were normally distributed they were not transformed. In order to conduct robust and coherent analyses of all measured metabolites nonparametric statistics were chosen. To select most body fat-relevant metabolites related with both WC (as aggregating measure of abdominal subcutaneous and visceral fat) and HC (as aggregating measure of gluteofemoral fat, muscles and bone structure), associations between metabolites and BMI (as marker of excess body fat and aggregating marker of body fat mass and lean mass) were first evaluated by means of Spearman's partial rank correlation coefficients (*r*). Correlation analyses were adjusted for height (per cm), age (per year), education (current in training/no certificate/skill; professional school; collage of higher education, university), smoking (never smoker; ex-smoker; smoker), physical activity (inactive; moderately inactive; moderately active; active), alcohol consumption (0 g per day; >0–6 g per day; >6–12 g per day; >12–24 g per day; >24–60 g per day; >60–96 g per day; >96 g per day), fasting status (fasting, non-fasting) and prevalent hypertension and were performed stratified by sex. As correlations of 127 metabolites with BMI, WC and HC showed almost identical relations with BMI and WC, but markedly lower relations with HC (data not presented), it appears unlikely that we missed relevant metabolites specifically related to abdominal and/or gluteofemoral body mass by the BMI-based selection process. Thereafter, metabolites with *r*⩾I0.20 I in men and/or women were analyzed regarding body shape. Median metabolite concentrations (95% confidence intervals) associated with abdominal fat were assessed across sex-specific tertiles of WC additionally adjusted for HC, whereas median metabolite concentrations associated with gluteofemoral body mass were assessed across sex-specific tertiles of HC additionally adjusted for WC. These statistical models with mutual adjustment of WC and HC allowed the investigation of metabolites related to abdominal fat mass (approximated through differing WC and constant HC) and gluteofemoral body mass (approximated through differing HC and constant WC). Further adjustments corresponded to the adjustment set described for the correlation analyses. The lowest tertile of metabolite concentrations were used as reference categories. Distribution-free 95% confidence intervals were calculated as described by Hahn and Meeker.^39^ The margin of I0.20 I was chosen to focus the analyses on metabolites of most metabolic relevance regarding abdominal and gluteofemoral body mass. This margin was considered as sufficiently high, as above this margin clearest trends across tertiles of abdominal and gluteofemoral body mass were observed.

All statistical analyses were carried out with SAS Enterprise Guide release 6.1 (SAS Institute, Cary, NC, USA). Statistical codes available from the corresponding author.

## Results

The present study population comprised 2270 adults (63.0% women) free of chronic diseases with a mean age of 49.7±8.9 years (mean±s.d.). Baseline characteristics are presented in Table 1. The study population was slightly overweight according to BMI. WC was at the upper boundaries of normal in both sexes.

Out of 127 metabolites a set of 23 metabolites showed *r*⩾I0.20 I with overall body mass, measured by BMI, in men and women, respectively (Table 2). This metabolite set included three AA (tyrosine, valine, isoleucine), five PCaa (C38:3, C38:4, C40:5, C40:6, C42:0), 11 PCae (C34:2, C34:3, C36:2, C38:2, C40:4, C42:3, C42:4, C42:5, C44:4, C44:5, C44:6), three lysoPC (C17:0, C18:1, C18:2) and one SM (C18:1). AA, the SM, and shorter-chained PCaa (⩽C40) were positively correlated with BMI, whereas PCae, lysoPCs and the longer-chained PCaa (C42) were negatively correlated. There were no differences in direction of correlations between men and women, but strength of correlations differed between sexes. Within the set of 23 metabolites, AA, PCaa, longer-chained PCae (C44) and lysoPC (C18) were stronger correlated in men, whereas the remaining PCae (⩽C42), lysoPC (C17) and the SM were stronger correlated in women. Significantly higher *r* were solely seen for tyrosine in men and PCae C38:2 in women.

**Figure 1:**
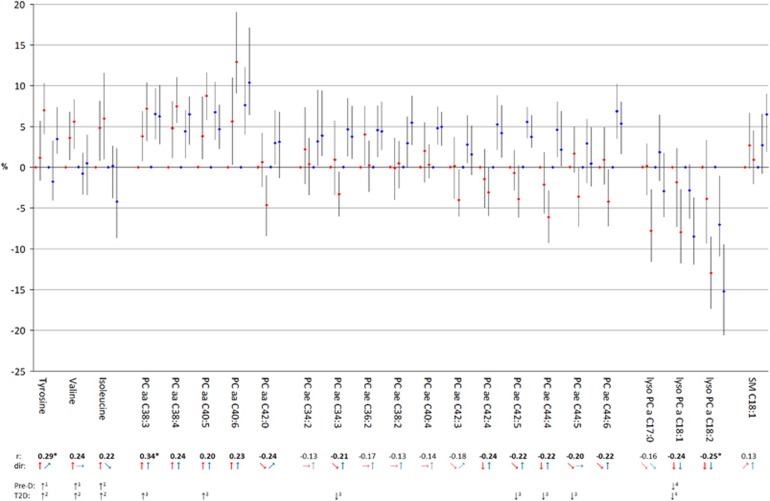
Median serum concentrations across tertiles of WC (red) and HC (blue) in relation to corresponding reference groups in men. Reference groups are lowest tertiles of WC and HC, respectively. Tertile classifications are as follows: WC tertile 1: WC<89 cm, WC tertile 2: 89 cm⩽WC<97 cm, WC tertile 3: WC⩾97 cm; HC tertile 1: HC<97 cm, HC tertile 2: 97 cm⩽HC< 102 cm, HC tertile 3: HC⩾102 cm. Adjustment sets included the following covariates: body height, age, education, smoking, physical activity, alcohol consumption, fasting status, prevalent hypertension as well as HC for concentrations across tertiles of WC and WC for concentrations across tertiles of HC, respectively. To gain a comprehensive view, correlation coefficients (*r*) with BMI are added below: *r*⩾I0.20 I are marked in bold, *r*⩾I0.25 I are additionally marked by asterisk. Directions (dir) of median serum concentrations across tertiles are presented by arrows: ↑ or ↓= clear trend; ↗ or ↘ =tendency; →= marginal tendency. Metabolites already associated with pre-diabetes (Pre-D) and/or type-2-diabetes (T2D) in previous studies are additionally marked: 1=Würtz *et al.*;^45^ 2=Wang *et al.;*^46^ 3=Floegel *et al.;*^24^ 4=Wang-Sattler *et al.*^21^ HC, hip circumference; WC, waist circumference.

**Figure 2:**
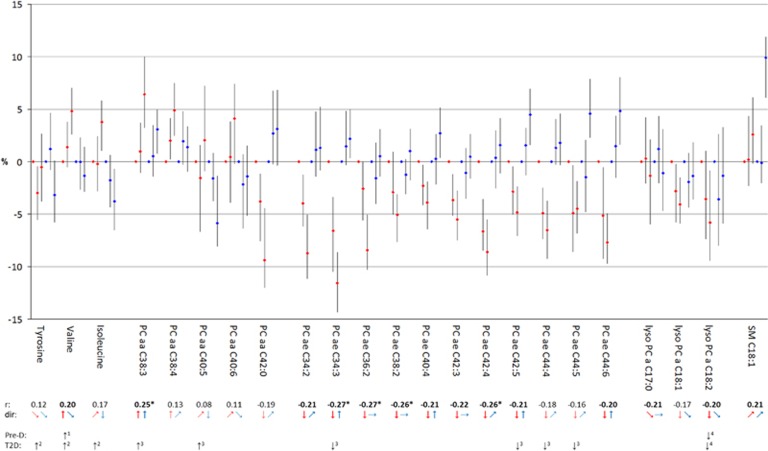
Median serum concentrations across tertiles of WC (red) and HC (blue) in relation to corresponding reference groups in women. Reference groups are lowest tertiles of WC and HC, respectively. Tertile classifications are as follows: WC tertile 1: WC<74 cm, WC tertile 2: 74 cm ⩽WC<83.5 cm, WC tertile 3:⩾83.5 cm; HC tertile 1: HC<97 cm, HC tertile 2: 97 cm⩽HC<103.5 cm, HC tertile 3: HC⩾103.5 cm. Adjustment sets included the following covariates: body height, age, education, smoking, physical activity, alcohol consumption, fasting status, prevalent hypertension as well as HC for concentrations across tertiles of WC and WC for concentrations across tertiles of HC, respectively. To gain a comprehensive view, correlation coefficients (*r*) with BMI are added below: *r*⩾I0.20 I are marked in bold, *r*⩾I0.25 I are additionally marked by asterisk. Directions (dir) of median serum concentrations across tertiles are presented by arrows: ↑ or ↓= clear trend; ↗ or ↘=tendency;→= marginal tendency. Metabolites already associated with pre-diabetes (Pre-D) and/or type-2-diabetes (T2D) in previous studies are additionally marked: 1=Würtz et al.;^45^ 2=Wang *et al.;*^46^ 3=Floegel *et al.;*^24^ 4=Wang-Sattler *et al.*^21^ HC, hip circumference; WC, waist circumference.

The set of 23 metabolites was thereupon analyzed regarding metabolite concentrations related to body shape. For each tertile of WC—adjusted for HC and other variables and thus reflecting abdominal fat—and HC—adjusted for WC and other variables and thus reflecting gluteofemoral body mass—, the median concentration of the selected metabolites was calculated. Figure 1 (men) and Figure 2 (women) show the results of these calculations for each metabolite illustrating the results for WC given a constant HC (WC_HC_) in red and the results for HC given a constant WC (HC_WC_) in blue. For the reader's convenience the direction of the medians across tertiles were additionally displayed by arrows below the graphics of the metabolite results. To gain a comprehensive view, correlations with BMI as well whether metabolites had been already identified as being associated with pre-diabetes and/or type-2-diabetes in previous studies are additionally indicated. We found that median metabolite concentrations across tertiles of WC_HC_ often show a similar pattern as the correlation of the metabolites with BMI. This similarity was most pronounced for metabolites with r⩾I0.20 I such as tyrosine, valine, isoleucine, PCaa C38:3, C38:4, C40:5, C40:6, PCae C42:4, C44:4, lysoPC C18:1 and C18:2 in men and valine, PCaa C38:3, PCae C34:2, C34:3, C36:2, C38:2, C40:4, C42:3, C42:4, C42:5, C44:6 and lysoPC C18:2 in women. Metabolites with r⩾I0.20 I and no clear trend across tertiles of WC_HC_ such as PCaa C42:0, PCae C34:3, C42:5, C44:5 and C44:6 in men and lysoPC C17 in women showed at least tendencies of concordant directions of associations, whereas metabolites with *r*<I0.20 I predominantly showed not more than tendencies across the tertiles. In contrast, median metabolite concentrations across tertiles of HC_WC_ were less concordant with the results of BMI and WC_HC_. This is of particular interest as associations with the analyzed metabolites were not studied before. Some metabolites showed median metabolite concentrations across tertiles of HC_WC_ in the same direction as across tertiles of WC_HC_, such as tyrosine, PCaa C38:3, C38:4, C40:5, C40:6, lysoPC C17:0, C18:1, C18:2 and SM C18:1 in men and tyrosine, PCaa C38:3, C38:4, lysoPC C18:1, C18:2 and SM C18:1 in women. However, some metabolites showed opposite directions as across tertiles of WC_HC_, such as isoleucine, PCaa C42:0, and PCae C34:3, C42:3, C42:4, C42:5, C44:4, C44:6 in men and valine, isoleucine, PCaa C40:5, C40:6, C42:0, PCae C34:2, C34:3, C40:4, C42:4, C42:5, C44:4, C44:5 and C44:6 in women. Moreover, some metabolites showed clear trends across tertiles of HC_WC_, but not more than tendencies across tertiles of WC_HC_, such as PCae C34:2, C36:2, C38:2, C40:4 in men; and some metabolites showed clear trends across tertiles of WC_HC_, but not more than tendencies across tertiles of HC_WC_, such as valine and PCae C44:5 in men and PCae C36:2, C38:2, C42:3 and lysoPC C17:0 in women. Consequently, the 23 metabolites could be subdivided into four groups: those being related in the same way with both measurements of body shape—the gluteofemoral body mass and the abdominal fat mass, those being inversely related with the measurements of body shape, and those being specifically related with either the gluteofemoral body mass or the abdominal fat mass. We noted that the assignment of the metabolites to one of the four groups is also sex-specific.

## Discussion

We analyzed 23 serum metabolites related to body mass with r⩾I0.20 I at least in one sex for their relations with body mass either located at the waist or at the hip. We observed that a body mass deposition at the hip has unique and sometimes opposite relations with metabolites compared with a deposition at the waist. The deposition at the waist shows similar relations with metabolites than overall fatness (BMI) and includes metabolites known to be related to type-2-diabetes.

First, we like to discuss the type of metabolites being considered as reflecting overall fatness that also includes specific deposition particularly at the waist. We identified two branched-chain (valine, isoleucine) and one aromatic (tyrosine) AA related to overall fatness. These results are in line with previous findings suggesting that both branched-chain (BCAA) and aromatic AA were positively associated with excess body weight.^16, 40^ Moreover, the observed stronger correlations in men basically support the hypothesis that males have higher rates of protein turnover than females.^16^ We could further show that shorter-chained PCaa (⩽C40) were positively correlated with overall fatness, and longer-chained PCaa (C42) and all PCae and lysoPC were negatively correlated. Thereby, PCaa were stronger related in men, whereas PCae were stronger related in women. In agreement with our findings lower PCae^41, 42^ and lower lysoPC concentrations were observed in obese individuals in other studies.^15, 17^ However, concerning lysoPC evidence is inconsistent as Pietiläinen *et al.*^41^reported higher lysoPC concentrations related to obesity. The present study also identified one SM (C18:1) related to overall body mass, conforming to previous results.^43^

The strong similarity of findings regarding metabolites associated with BMI and WC_HC_ is not surprising as BMI and WC are highly correlated. This confirms a strong positive correlation between the underlying metabolite networks of BMI and WC (*r*=0.99) within the same data set.^44^ This finding is also supporting our decision to select metabolites for further analysis based on correlations with BMI and on the margin of I0.20 I. Above this margin, clearest trends of median metabolite concentrations across tertiles of abdominal and gluteofemoral body mass were observed. Metabolites specifically related to abdominal fat were observed for different metabolites in men (valine, PCae C44:5) and women (PCae C36:2 C38:2, C42:3, lysoPC C17:0). Interestingly, the two metabolites observed in men were previously identified as risk metabolites of pre-diabetes^45^ and type-2-diabetes.^24, 46^ They might act as early indicators of adverse health implications specifically related to abdominal fat. The exact mechanism that explains elevated health risks associated with WC is not firmly established. It is assumed that visceral fat has an important etiological role of diabetes by release of free fatty acids directly from visceral fat into hepatic circulation, resulting in insulin resistance, hyperinsulinemia,^27, 28^ increased gluconeogenesis and dyslipidemia.^47, 48^ So far, little attention has been given to associations between type of body shape and metabolite profiles in metabolically healthy individuals. The revealed metabolites are possibly involved in underlying mechanisms resulting in such disorders. Increased protein catabolism secondary to insulin resistance has been suggested as possible explanation for increased BCAA in obesity.^49^ In moderate upper body obesity increased proteolysis and impairment of insulin's antiproteolytic action was found.^50, 51^ Furthermore, tissue-specific alterations in BCAA metabolism, in the liver and adipose tissue but not in muscle, may contribute to increased plasma concentrations of BCAA in obesity.^49^ Besides higher serum concentrations of BCAA we found lower serum concentration of PCae and lysoPC to be related with increased abdominal fat. Plasma phospholipids, mostly secreted by the liver, are abundantly present in all classes of lipoproteins. PCae were found to be inversely correlated with plasma triglycerides.^24^ Furthermore, higher levels of PCae were observed to be related with improved insulin sensitivity and reduced insulin secretion;^24^ in addition, they may act antioxidative and prevent lipoprotein oxidation.^42^

The novel insight relates to the body mass at the hip—mostly reflecting subcutaneous fat, muscle mass and bone structure (pelvic width). It seems as if increased body mass at this site has its own metabolic consequences, probably with less-adverse implications than large overall fat volumes and abdominal fat when the metabolites related to this deposition were taken into consideration. The interpretation of metabolic consequences related to body mass at the hip may vary between sexes. Although gluteofemoral fat and pelvic width may be the main components of HC in women, pelvic width and muscles may be the main components in men. On the one hand, underlying mechanisms of less-adverse implications may be related to subcutaneous fat, which has lower levels of basal lipolysis and lipolytic stimulation compared with visceral fat, potentially resulting in lower flux of free fatty acids into the blood.^47^ Furthermore, it has been suggested that subcutaneous fat may act as a sink for circulating free fatty acids.^48^ Subcutaneous fat is additionally supposed to consist of smaller adipocytes that have relatively higher insulin sensitivity.^52^ Owing to differences in body fat distribution metabolic impact of subcutaneous fat may vary between men and women. On the other hand, less-adverse implications may be related to muscle mass, the main target organ for insulin and one of the sites of insulin resistance.^30^ A lower HC possibly indicates relatively small gluteal and leg muscle mass. This could be more apparent in men owing to lower gluteofemoral fat storage compared with women. It has been shown in healthy men that relatively small leg muscle mass is related to an elevated waist-to-hip ratio^53^ and to different glucose levels between Indian and Swedish men.^54^

We could note that also deposition of body mass at this site show for some metabolites similar relations with overall and abdominal fat. Those metabolites showing corresponding associations in men and women included tyrosine, PCaa C38:3, C38:4, lysoPC C18:1, C18:2, and SM C18:1—with mostly clearer trends in men. We also observed some metabolites related in men only (PCaa C40:5, C40:6, lysoPC C17:0). Tyrosine, PCaa C38:3, C40:5 and lysoPC C18:2 were already found to be related to pre-diabetes and/or type-2-diabetes.^21, 24, 45, 46^ Metabolites with concordant relations with abdominal and gluteofemoral body mass might be considered as reflecting large body volumes and include indicators of elevated metabolic risks associated with excess body weight in general wherever it is located. Higher levels of aromatic AA have been related to excess body fat, insulin^55^ and insulin resistance.^12^ Choline-containing phospholipids such as PCaa and lysoPC represent the major components of cellular membranes.^56^ Their blood concentration may be influenced by hepatic *de novo* synthesis and redistribution from plasma membranes.^57^ As constituents of lipoproteins, PCaa have been related to hepatic secretion of triyglceride-rich VLDL particles and HDL.^56^ The observed associations of sphingomyelin SM C18:1 with higher abdominal and gluteofemoral body mass seem to be attributed to both fat and muscle mass, what is in line with previous studies.^58, 59^

However, we also observed metabolites showing opposite associations regarding type of body shape. In men and women, isoleucine, PCaa C42:0, PCae C34:3, C42:4, C42:5, C44:4 and C44:6 exhibited this property—with mostly clearer trends in women. We also observed some metabolites related in women only (valine, PCaa C40:5, C40:6, PCae C34:2, C40:4, C44:5). Valine, isoleucine, PCaa C40:5, PCae C34:3, C42:5, C44:4 and C44:5 were already found to be related to pre-diabetes and/or type-2-diabetes.^24, 45, 46^ To our surprise, the direction of relations with the compartment ‘waist' corresponded to the findings with pre-diabetes and/or type-2-diabetes, and the relations with the compartment ‘hip' were opposite. This finding strengthens the view that gluteofemoral body mass deposition might even induce metabolic processes protecting against type-2-diabetes. In women, fat deposition at the hip region and regional differences in adipocyte metabolism (lipoprotein lipase activity and lipolysis) are more pronounced than in men.^60^ In the present study a possible protective role of lager hips was found to be more apparent in women than in men. This finding suggests that metabolic processes protecting against type-2-diabetes might be particularly linked to subcutaneous fat deposition.

Metabolites specifically related to gluteofemoral body mass only were solely observed in men (PCae C34:2, C36:2, C38:2, C40:4) and comprised none of the identified risk metabolites for pre-diabetes and/or type-2-diabetes. The present findings suggest that gluteofemoral body mass deposition has different metabolic consequences and support the hypothesis that increased gluteofemoral body mass presumably goes along with less-adverse health implications.

Among the strengths of the present study are the use of standardized procedures for the assessment of anthropometry by trained personnel and the availability of concentrations of metabolites with known identity comprising different classes. Determination by the validated Absolute*IDQ* p150 kit allowed simultaneous quantification using a modern high-throughput technique, which has been approved and standardized. Further strengths of our study are the possibility to consider relevant confounding variables and the exclusion of individuals with chronic diseases to avoid effects of accompanying metabolic changes. The limitations of the study refer to its cross-sectional nature and the use of anthropometrical measurements, which are only rough estimations of the true body composition. Measurements of body composition obtained by direct methods such as dual-energy X-ray absorptiometry, magnetic resonance imaging or bodyscanner accurately and precisely quantify compartments of body composition. Such methods require special and expensive equipment and are often unsuitable to screen large samples. Indubitably, mutual adjustment of WC and HC can only provide an approximation of metabolite concentration differences related to abdominal and gluteofemoral body mass, respectively. Nevertheless, to receive hints for differences in associations related to abdominal and gluteofemoral body mass, it seems methodologically appropriate. A further limitation relates to the selection of metabolites, which is highly artificial and of rather small extent when considering what's technically possible in metabolomics. Furthermore, variances of metabolite concentrations depend not only on biological variability, but also on the precision of the measurements, which might have influenced our assumption of metabolic relevance. Based on findings of a reliability study, metabolites below the detection limit and those with high analytical variance were excluded to ensure valid measurements.^38^

In conclusion, the study confirmed several metabolite associations with BMI as anthropometric marker of overall body mass, provided metabolic profiles concerning WC_HC_ as marker of abdominal fat mass and revealed specific metabolic profiles for HC_WC_ as marker of gluteofemoral body mass differing from those of overall body mass and abdominal fat. Thereby, we observed sex-specific differences. Metabolites already identified as being associated with pre-diabetes and/or type-2-diabetes were assignable to the three abdominal fat-related metabolite groups and appear independent from their associations with gluteofemoral body mass, underscoring the lower adverse metabolic consequences by this compartment. The observation of independent associations of metabolites with gluteofemoral body mass as well as opposite relations with abdominal and gluteofemoral body mass even suggest that increased deposition in the gluteofemoral area could possibly reduce the risk of type-2-diabetes owing to beneficial metabolic impacts. Future studies using direct measurements of body composition need to further validate these findings.

## Figures and Tables

**Table 1:** Baseline characteristics of the study population

*Baseline characteristics*	*Total*	*Men*	*Women*
	*(*n=*2270)*	*(*n=*839)*	*(*n=*1431)*
*Anthropometry*			
BMI (kg m^−2^)	25.9 (4.1)	26.5 (3.4)	25.5 (4.5)
Waist circumference (cm)	85.0 (12.5)	93.4 (9.7)	80.1 (11.2)
Hip circumference (cm)	100.6 (7.9)	99.7 (6.0)	101.1 (8.8)

*Education*			
Current in training/no certificate/skill	36.74	31.35	39.90
Professional school	24.58	15.38	29.98
Collage of higher education, university	38.68	53.28	30.12

*Smoking status*			
Never smoker	47.62	29.92	58.00
Ex-smoker	31.54	43.86	24.32
Smoker	20.84	26.22	17.68

*Physical activity*			
Inactive	19.38	18.24	20.06
Moderately inactive	39.60	38.26	40.39
Moderately active	24.01	24.91	23.48
Active	17.00	18.59	16.07

*Alcohol consumption*			
0 g per day	2.78	2.98	2.66
>0 to 6 g per day	38.85	16.33	52.06
>6 to 12 g per day	20.97	15.97	23.90
>12 to 24 g per day	18.11	24.79	14.19
>24 to 60 g per day	16.34	32.78	6.71
>60 to 96 g per day	2.47	5.84	0.49
>96 g per day	0.48	1.31	—

Continuous variables are presented as means (s.d.). Categorical variables are presented in percent. Anthropometric variables and covariates are presented in total and separated by sex. Alcohol consumption category >96 g per day includes only men.

**Table 2:** Spearman's partial rank correlation coefficients *r* (95% CI) of serum metabolites with BMI, stratified by sex

*Metabolites*	*Men*	*Women*
	*(*n=*839)*	*(*n=*1431)*
*Amino Acids*		
Tyrosine*	**0.29** (0.22; 0.35)	0.12 (0.07; 0.17)
Valine	**0.24** (0.17; 0.30)	**0.20** (0.15; 0.25)
Isoleucine	**0.22** (0.15; 0.28)	0.17 (0.12; 0.22)

*Diacyl-phosphatidylcholines*		
PCaa C38:3*	**0.34** (0.27; 0.39)	**0.25** (0.20; 0.30)
PCaa C38:4	**0.24** (0.17; 0.30)	0.13 (0.08; 0.18)
PCaa C40:5	**0.20** (0.13; 0.27)	0.08 (0.02; 0.13)
PCaa C40:6	**0.23** (0.16; 0.29)	0.11 (0.06; 0.16)
PCaa C42:0	**−0.24** (−0.30; −0.17)	−0.19 (−0.24; −0.14)

*Acyl–alkyl-phosphatidylcholines*		
PCae C34:2	−0.13 (−0.20; −0.06)	**−0.21** (−0.26; −0.16)
PCae C34:3*	**−0.21** (−0.27; −0.14)	−**0.27** (−0.31; −0.22)
PCae C36:2*	−0.17 (−0.23; −0.10)	−**0.27** (−0.32; −0.22)
PCae C38:2*	−0.13 (−0.20; −0.06)	−**0.26** (−0.31; −0.22)
PCae C40:4	−0.14 (−0.21; −0.07)	−**0.21** (−0.26; −0.16)
PCae C42:3	−0.18 (−0.25; −0.11)	−**0.22** (−0.27; −0.17)
PCae C42:4*	−**0.24** (−0.30; −0.17)	−**0.26** (−0.30; −0.21)
PCae C42:5	−**0.22** (−0.28; −0.15)	−**0.21** (−0.26; −0.16)
PCae C44:4	−**0.22** (−0.28; −0.15)	−0.18 (−0.23; −0.13)
PCae C44:5	−**0.20** (−0.27; −0.13)	−0.16 (−0.21; −0.10)
PCae C44:6	−**0.22** (−0.28; −0.15)	−**0.20** (−0.25; −0.15)

*Lyso-phosphatidylcholines*		
lysoPC a C17:0	−0.16 (−0.23; −0.10)	−**0.21** (−0.26; −0.16)
lysoPC a C18:1	−**0.24** (−0.30; −0.17)	−0.17 (−0.22; −0.12)
lysoPC a C18:2*	−**0.25** (−0.31; −0.19)	−**0.20** (−0.25; −0.15)

*Sphingomyelins*		
SM C18:1	0.13 (0.06; 0.20)	**0.21** (0.16; 0.26)

Abbreviations: a, acyl; aa, diacyl; ae, acyl–alkyl; BMI, body mass index; CI, confidence interval; PC, phosphatidylcholine; SM, sphingomyelin. Correlation coefficients are adjusted for body height, age, education, smoking, physical activity, alcohol consumption, fasting status and prevalent hypertension, and rounded to two decimal places. Correlation coefficients ⩾I0.20 I are marked in bold. Metabolites with correlation coefficients ⩾I0.25 I in men and/or women are additionally marked by asterisks (*). Fatty-acid side chains were abbreviated C*x*:y where *x* represents the number of carbon atoms and y the number of double bounds.
